# Toward More Cost-Effective Hydrogen Production: Magnetron-Sputtered Ni- and Co-Based CeO_2_ Catalysts at Ultralow Metal Loading

**DOI:** 10.1021/acsomega.6c06081

**Published:** 2026-10-14

**Authors:** Leticia Forrer Sosa, Marco Aurélio Suller Garcia, Adriano Friedrich Feil, Dario Eberhardt, Santiago José Alejandro Figueroa, Carlos Alberto Ospina Ramirez, Angela Albuquerque, João Monnerat Araújo Ribeiro de Almeida, Pedro Nothaft Romano

**Affiliations:** † Instituto de Química, 28125 Universidade Federal do Rio de Janeiro (UFRJ), Rio de Janeiro, Rio de Janeiro 21941-909, Brazil; ‡ LIPCAT (Laboratório de Intensificação de Processos e Catálise), UFRJ, Rio de Janeiro, Rio de Janeiro 21941-594, Brazil; § Nanotechnology Engineering Program, Alberto Luiz Coimbra Institute for Graduate Studies and Research in Engineering, COPPE, UFRJ, Rio de Janeiro, Rio de Janeiro 21941-972, Brazil; ∥ BlueNano, TECNOPUC, Porto Alegre, Rio Grande do Sul 90610-001, Brazil; ⊥ Brazilian Synchrotron Light Laboratory (LNLS), Centro Nacional de Pesquisa em Energia e Materiais (CNPEM), Campinas, São Paulo 13083-100 Brazil; # Institute of Chemistry, University of Campinas, Campinas, São Paulo 13083-862, Brazil; ∇ Brazilian Nanotechnology National Laboratory, 215006 Brazilian Center for Research in Energy and Materials, Campinas, São Paulo 13083-100, Brazil

## Abstract

A series of mono- and bimetallic Ni- and Co-based catalysts supported on nanocrystalline cerium oxide (CeO_2_) was prepared by magnetron sputtering with a total metal loading of only 0.2 wt % to investigate the influence of the deposition sequence on the ethanol steam reforming (ESR) reaction. Structural analyses by X-ray Diffraction (XRD) and Scanning Transmission Electron Microscopy-Energy-Dispersive X-ray Spectroscopy (STEM-EDS) confirmed that the sputtering process produced highly dispersed metallic species without detectable crystalline domains, while maintaining the mesoporous structure of the CeO_2_ support. Among the catalysts, Ni–Co/CeO_2_, obtained by sequential deposition of Co followed by Ni, exhibited the lowest deactivation rate constant (kd = 9.93 × 10^–3^ h^–1^), highest stability, and H_2_ selectivity sustained over 28 h of reaction. Postreaction characterization by EDS mapping, Thermogravimetric Analysis-Derivative Thermogravimetry (TGA-DTG), and Raman spectroscopy revealed minimal coke deposition on Ni–Co/CeO_2_, whereas the Co–Ni and NiCo counterparts suffered from higher carbon accumulation and faster deactivation. These results demonstrate that the metal deposition order critically governs the catalytic performance and that magnetron sputtering enables precise surface control even at ultralow metal loadings, producing catalysts with remarkable activity, stability, and coke resistance. Thus, herein, we highlight that magnetron sputtering is a promising route for designing efficient and economically viable reforming catalysts, which opens perspectives for exploring electronic and interfacial effects at higher metal loadings.

## Graphical abstract

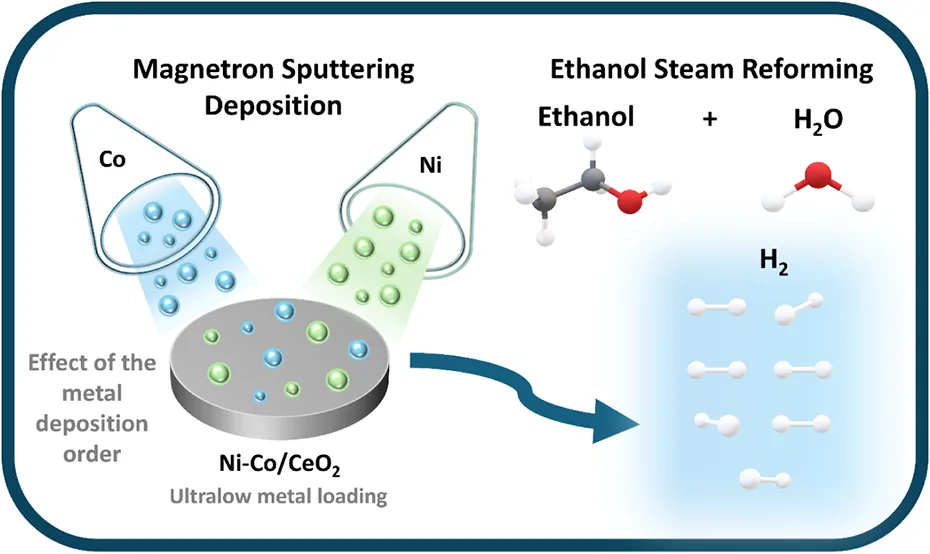

## 1 Introduction

The transition toward a low-carbon energy matrix requires sustainable routes for hydrogen production that can integrate renewable resources while minimizing greenhouse gas emissions. Ethanol steam reforming (ESR) has attracted significant interest in this context, particularly in regions with established bioethanol industries, such as Brazil.^1–3^ Ethanol is a renewable, carbon-neutral feedstock that can be obtained from biomass and utilized in distributed reforming units to generate hydrogen with reduced environmental impact compared to fossil-based routes.^4,5^ However, the successful deployment of ESR technologies depends on the design of highly active, selective, and stable catalysts capable of suppressing byproduct formation and mitigating carbon deposition.

Nickel-based catalysts are widely employed in ESR due to their high intrinsic activity toward C–C and O–H bond activation.^6–8^ Nevertheless, Ni systems often suffer from rapid deactivation, primarily through coke accumulation and sintering under reforming conditions.^6^ On the other hand, cobalt-based catalysts, especially over certain supports, such as ceria (CeO_2_), display improved oxygen mobility and enhanced resistance to carbon deposition, though typically at the expense of lower hydrogen selectivity and a higher tendency toward undesired side reactions.^9^ CeO_2_ is a well-established support in reforming catalysis owing to its remarkable redox flexibility between Ce^3+^ and Ce^4+^ states, which enables oxygen vacancy formation and lattice oxygen mobility.^10^ These properties not only enhance the dispersion and stabilization of transition metals but also contribute to coke gasification during the reaction.

Within this scenario, the combination of Ni and Co in bimetallic configurations over CeO_2_ has emerged as a promising strategy to exploit synergistic effects, where Ni provides high reforming activity and Co promotes redox functionality.^2,11–13^ Still, the precise relationship between metal arrangement and resistance to coke remains underexplored. Although studies have shown that sequential or co-impregnation of Ni–Co on supports such as alumina, ceria, or mixed oxides can influence dispersion and stability, little work has addressed this issue using magnetron-sputtered Ni–Co systems.^14–16^ This technique allows for precise control over metal distribution at the nanoscale, enabling the exploration of sequence-controlled deposition strategies that may fundamentally alter the physicochemical properties of the resulting materials.^17–19^

In this work, we investigate mono- and bimetallic Ni- and Co-based catalysts supported on nanocrystalline CeO_2_, prepared by magnetron sputtering with different deposition sequences. Comprehensive characterization by XRD, high-resolution transmission electron microscopy (HRTEM), STEM-EDS, H_2_ chemisorption, temperature-programmed reduction (TPR), and X-ray photoelectron spectroscopy (XPS) was employed to correlate structural and electronic properties with ESR performance. We show that the sequential deposition of Co followed by Ni (Ni–Co/CeO_2_) yields superior resistance to carbon accumulation and enhanced hydrogen selectivity over 28 h of reaction, outperforming both monometallic and other bimetallic configurations. Thus, we could establish the deposition sequence as a critical design parameter for engineering and catalyst optimization in ESR, offering valuable insights into the development of efficient and durable hydrogen production systems.

## 2 Experimental Section

### 2.1 Catalysts’ Preparation

Mono- and bimetallic catalysts with a nominal loading of 0.2 wt % were synthesized on a CeO_2_ (nanopowder, <25 nm, Sigma-Aldrich) support by magnetron sputtering deposition. The monometallic materials contained either Ni or Co, whereas the bimetallic compositions were produced with equal mass contributions. To ensure homogeneous coverage during deposition, approximately 3 g of CeO_2_ powder was placed in an aluminum container mounted on a mechanical resonant agitator operating at 23 Hz inside the sputtering chamber. The chamber was first evacuated to 2 × 10^–7^ mbar and subsequently filled with high-purity Ar (99.999%) until reaching a working pressure of 4 × 10^–3^ mbar. Deposition was carried out under 50 W DC power using high-purity Ni and/or Co metallic targets (99.99%, 2″ diameter). The catalysts were named according to the active phase: Ni/CeO_2_ and Co/CeO_2_ for the monometallic systems. For bimetallic samples, three distinct deposition sequences were tested: (i) sequential deposition of Co followed by Ni (Ni–Co/CeO_2_), (ii) the reverse sequence (Co–Ni/CeO_2_), and (iii) simultaneous deposition of both metals (NiCo/CeO_2_).

### 2.2 Catalyst Characterization

Textural parameters were determined by N_2_ adsorption–desorption at 77 K on an Autosorb iQ analyzer (Anton-Paar, Graz, Austria). Before the measurements, the samples were degassed at 300 °C under vacuum for 18 h. The specific surface area was obtained from the Brunauer–Emmett–Teller (BET) method, while pore volume and pore size distribution were calculated by the Barrett–Joyner–Halenda (BJH) model.

X-ray diffraction (XRD) patterns were acquired on an AXRD LPD diffractometer (Proto, Michigan, USA), using Cu Kα radiation (40 kV, 40 mA). Data were collected in the 2θ range of 20–100° with a step size of 0.02° and an acquisition time of 1 s per step. Reducibility and surface hydrogen uptake were probed by H_2_ temperature-programmed reduction (TPR) and chemisorption on an AutoChem II system (Micromeritics). In these experiments, 0.2 g of catalyst was pretreated in Ar (50 mL min^–1^) at 150 °C (heating rate 10 °C min^–1^) for 30 min. After cooling to 50 °C, the gas flow was switched to 10 (v/v) % H_2_/Ar (50 mL min^–1^), and the sample was heated to 500 °C. For H_2_ chemisorption, the reduced catalyst was cooled to 40 °C, purged with Ar for 30 min, and then exposed to calibrated 10 (v/v) % H_2_/Ar (50 mL min^–1^) pulses until saturation. Surface chemical states were examined by X-ray photoelectron spectroscopy (XPS) using a Thermo Scientific K-Alpha instrument (Massachusetts, USA), with a microfocused monochromatic Al Kα source (1486.68 eV, 12 kV, 72 W). Data were recorded at pass energies of 150 eV for survey scans and 20 eV for high-resolution scans with 1 and 0.1 eV step sizes, respectively. Charge neutralization of the samples was achieved using an FG03 flood gun (electron + ion source). Transmission-corrected data were analyzed in CasaXPS software (v2.3.27PR4.4) using a Shirley-type background and Scofield cross sections. Ce 3d peaks were fitted using the informed sample model approach, as proposed by Morgan.^20^

The morphology and nanoscale features of the fresh catalysts were observed by transmission electron microscopy (TEM) on a double spherical aberration-corrected Titan Cubed Themis 80–300 (FEI Company/Thermo Fisher Scientific, USA) operated at 300 kV and equipped with a Super-X EDS detector system. Bright-field and high-angle annular dark-field scanning TEM (BF/HAADF-STEM) detection/illumination modes were employed. For sample preparation, 0.01 g of catalyst was ultrasonically dispersed in 3 mL of isopropyl alcohol for 10 min, and a drop of the suspension was deposited on a 400-mesh copper grid with lacey and ultrathin carbon layers. The morphology of spent samples was additionally investigated by scanning electron microscopy (SEM) using a JSM-IT700HR microscope (JEOL, Tokyo, Japan) following the same preparation procedure.

Coke deposition was quantified by thermogravimetric analysis (TGA) on an SDT 650 Discovery system (TA Instruments, New Castle, USA). Measurements were carried out with 0.030 g of sample, heated from 30 to 1000 °C at 5 °C min^–1^ under air flow (100 mL min^–1^). Raman spectroscopy was further used to probe the structural features, employing an Xplora Plus microscope (Horiba, Kyoto, Japan) with a 532 nm excitation source. Spectra were collected from 100 to 1000 cm^–1^ using a 100× objective, a 1200 g/mm grating, an acquisition time of 15 s per scan, five accumulations, and a 1% neutral density filter.

### 2.3 Catalytic Evaluation

Catalytic tests for ethanol steam reforming (ESR) were performed in a stainless-steel fixed-bed tubular reactor operated at atmospheric pressure and charged with 0.2 g of catalyst. Before the reaction, the catalysts were subjected to *in situ* reduction under a 25 (v/v) % H_2_/N_2_ stream (100 mL min^–1^) by heating to 500 °C at a rate of 5 °C min^–1^. Once reduced, the gas was switched to pure N_2_ (50 mL min^–1^), and an aqueous ethanol solution (ethanol-to-water molar ratio = 1:6) was delivered at 0.06 mL min^–1^ using a high-performance liquid chromatography (HPLC) pump. The liquid feed was vaporized at 200 °C before entering the catalytic bed. The reaction conditions corresponded to a weight-to-feed ratio (W/F) of 0.2 g_cat_ h g_EtOH_^–1^.

Product gases were monitored online using a Trace 1610 gas chromatograph (Thermo Scientific) fitted with both a flame ionization detector (FID) and a thermal conductivity detector (TCD). Gas separation was achieved with an Rt-Q BOND column (30 m × 0.32 mm × 10 μm) connected to the FID, and HayeSep Q (2 m × 2 mm, 80/100 mesh) and Molesieve 13X (2 m × 1/8 in., 80/100 mesh) columns connected to the TCD. The condensed liquid products were analyzed by HPLC (Dionex UltiMate 3000, Thermo Scientific) equipped with a refractive index detector (RID), a Micro-Guard Cation H^+^ guard column (30 × 4.6 mm, Bio-Rad), and an Aminex HPX-87H column (300 × 7.8 mm, Bio-Rad).

Ethanol conversion, product selectivity, and the hydrogen production rate (HPR) were calculated according to eqs 1–3:

1 $$EtOH\;conversion\;\left(\%\right)=\frac{mol_{EtOH}^{0}-mol_{EtOH}^{t}}{mol_{EtOH}^{0}}\times 100$$

2 $$Product\;distribution\;\left(\%\right)=\frac{mol_{EtOH}^{t}}{\overset{N}{\underset{i=0}{\sum }}mol_{i}^{t}}\times 100$$

3 $$HPR\left(L/\mathrm{h.}g_{cat}\right)=\frac{mol_{EtOH}^{0}xY_{H2}x6x22.4}{m_{cat}x{\%}_{metal}}\times 100$$

where *mol* ^0^_*EtOH*_ and *mol^t^*_*EtOH*_ are the molar flow rates of ethanol in the feed and at any time *t*, respectively; *mol^t^_i_* are the molar flow rates of product *i* at any time *t*. *Y*_*H*2_ is the yield of hydrogen.

## 3 Results and Discussion

### 3.1 Physicochemical Characterizations of the Prepared Catalysts

The mono- and bimetallic Ni- and Co-based catalysts were synthesized by magnetron sputtering onto a commercial nanocrystalline CeO_2_ support, aiming for a uniform dispersion of the active phase; the procedure was performed to allow a 0.2 wt % metal loading in each catalyst. The XRD patterns of the catalysts were the first data obtained (Figure S1) and exhibited only the reflections characteristic of the cubic fluorite structure of the CeO_2_ support (2θ = 28.5°, 33.1°, 47.5°, 56.3°, JCPDS card #34–0394),^21^ without detectable signals from metallic Ni, Co, or their oxide counterparts. Such results are consistent with the low loading aimed for and a high dispersion of the nanoparticles over the support, which hindered the formation of large crystalline domains. ICP-OES analysis confirmed that the metal contents were consistent with the targeted compositions. The monometallic catalysts contained 0.2 wt % of the corresponding metal (Ni or Co), whereas the bimetallic catalysts contained 0.1 wt % Ni and 0.1 wt % Co, resulting in a total metal loading of 0.2 wt % in all samples. These results confirm that the intended metal loadings were successfully achieved during the magnetron sputtering deposition. Nevertheless, ICP-OES provides bulk compositional information and does not reveal the spatial distribution, dispersion, or morphology of the deposited metallic species. Therefore, electron microscopy analyses were subsequently performed to investigate the morphology and nanoscale distribution of Ni and Co over the CeO_2_ support.

Figure 1 shows the structural and compositional characterization of the series of catalysts supported on CeO_2_ obtained by BF/HAADF-STEM and STEM-EDS elemental mapping. In the BF-STEM images (first column of each set), the CeO_2_ support exhibits sharp edges and well-defined lattice fringes. However, distinct Ni or Co nanoparticles or clusters are not readily discernible in any of the catalysts, irrespective of the deposition sequence or metal composition. This observation is attributed to the ultralow total metal loading (0.2 wt %) and the high dispersion of the deposited species, which makes the metallic domains difficult to resolve against the CeO_2_ support in BF-STEM images. Additionally, HAADF is sensitive to mass/thickness as well as Z; thus, Co and Ni nanoparticles or very small clusters deposited on CeO_2_ can be essentially “masked” in the much stronger Ce signal. Furthermore, Ni (Z = 28) and Co (Z = 27) are neighboring elements and consequently exhibit similar electron-scattering contrast, making their differentiation based solely on BF-STEM contrast particularly challenging.

**Figure 1:**
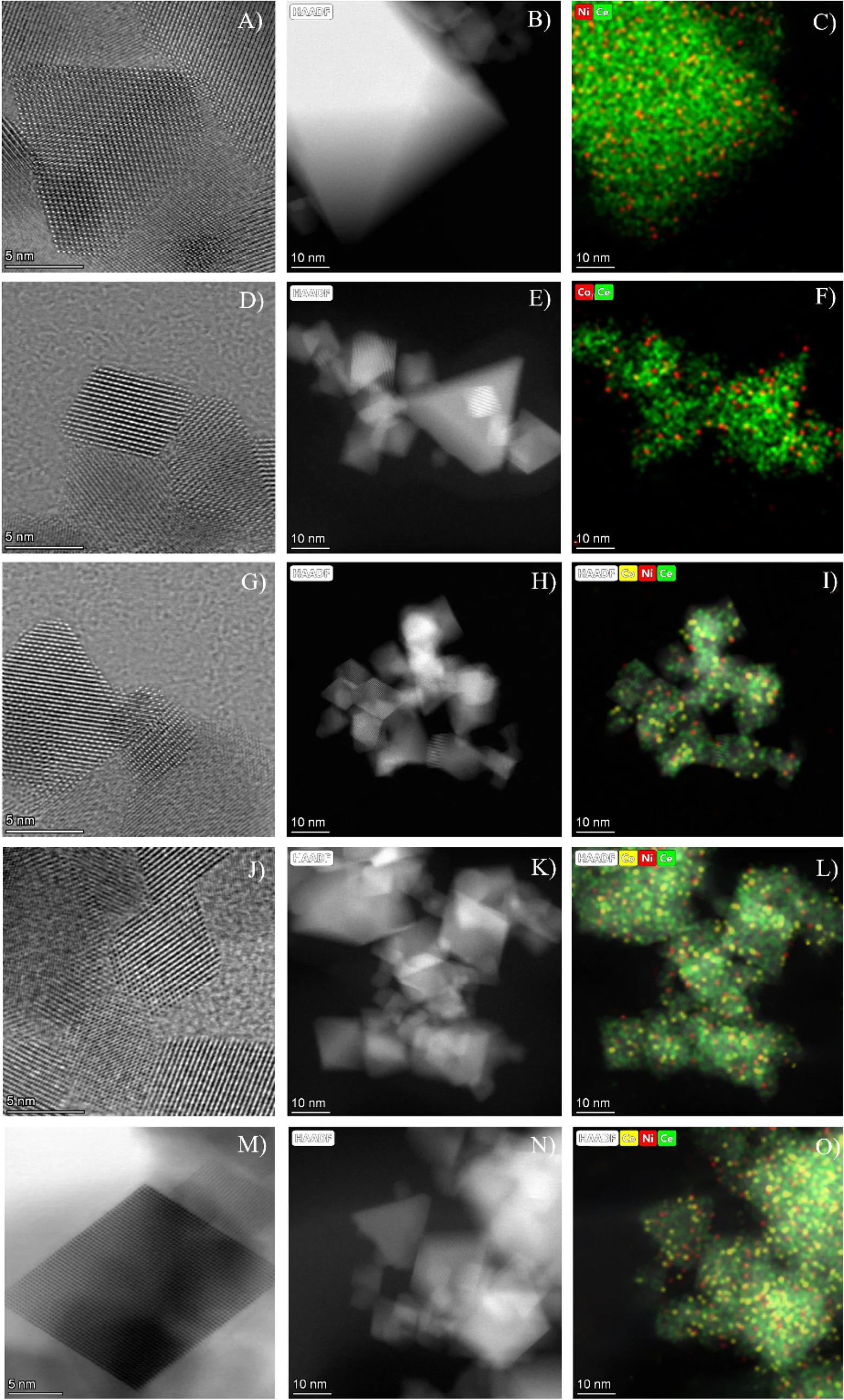
A, D, G, J, M) BF-STEM, B, E, H, K, N) HAADF-STEM, and C, F, I, L, O) STEM-EDS elemental mapping images of CeO_2_-supported catalysts: A–C) Ni/CeO_2_, D–F) Co/CeO_2_, G–I) NiCo/CeO_2_, J–L) Ni–Co/CeO_2_, and M–O) Co–Ni/CeO_2_.

Thus, HAADF-STEM images (second column of each set) were acquired and used to obtain the STEM-EDS elemental maps (third column of each set) to confirm the spatial distributions of Co, Ni, and Ce. For the Ni/CeO_2_ catalyst (Figure 1A–C), Ni (red) appears uniformly spread without forming large segregated clusters (Figure 1C); for the monometallic Co/CeO_2_ catalyst (Figure 1D–F), red Co signals are also homogeneously distributed over the green CeO_2_ matrix (Figure 1F). In the bimetallic samples (Figure 1I, 1L, and 1O), both Ni and Co signals overlap extensively with Ce, suggesting intimate contact with the support surface. However, no clear differences in the spatial distribution or colocalization of Ni and Co can be identified between the different deposition sequences from these maps alone. Therefore, the STEM-EDS results confirm the presence and distribution of both metals but do not provide sufficient spatial resolution or compositional sensitivity to establish subtle differences in their surface arrangements arising from the deposition sequence. Nevertheless, taken together, these results indicate that the magnetron sputtering synthesis strategy employed led to highly dispersed metal species without the formation of large nanoparticles.

Next, the data in Table 1 provide insights into the textural properties obtained from N_2_ physisorption and the surface characteristics derived from H_2_ chemisorption for the metal-modified catalysts. The specific surface areas range from 55 to 66 m^2^ g^–1^, with the highest values observed for Co-containing (66 m^2^ g^–1^) and Ni–Co bimetallic catalysts (65 m^2^ g^–1^), while the NiCo sample presents the lowest value among the modified materials (55 m^2^ g^–1^). The pore volumes vary between 0.20 and 0.42 cm^3^ g^–1^: Co-based catalysts display relatively high values (0.42 cm^3^ g^–1^), whereas the NiCo bimetallic sample shows the lowest (0.20 cm^3^ g^–1^). Regarding pore diameter, the catalysts exhibit values in the range of 2.9–3.4 nm. The adsorption–desorption N_2_ isotherms of the mono- and bimetallic catalysts are presented in Figure S2. All samples display type IV isotherms, characteristic of mesoporous materials.^22^

**Table 1:** Chemical Composition, Textural Properties, and H_2_ Chemisorption of Mono- and Bimetallic Ni/Co Catalysts

	N_2_ physisorption			H_2_ chemisorption		XPS	
Material	S_BET_ (m^2^ g^–1^)	Vp (cm^3^ g^–1^)	Dp (nm)	S_M_ (m^2^ g_cat_^–1^)	Dispersion (%)	Ce^3+^	Ce^4+^
Ni/CeO_2_	63	0.25	3.1	0.30	4	15	85
Co/CeO_2_	66	0.42	3.2	1.00	12	16	84
NiCo/CeO_2_	55	0.20	3.4	0.74	11	16	84
Ni–Co/CeO_2_	65	0.36	2.9	1.22	18	15	85
Co–Ni/CeO_2_	59	0.25	3.4	0.87	13	16	84

Hydrogen chemisorption results highlight clear differences in the metallic surface area (S_M_) and dispersion. Among the monometallic catalysts, Ni/CeO_2_ exhibits a very low S_M_ (0.30 m^2^ g_cat_^–1^) and poor dispersion (4%). In contrast, Co/CeO_2_ shows markedly higher values (S_M_ = 1.00 m^2^ g_cat_^–1^, dispersion = 12%). Ni–Co/CeO_2_ stands out with the highest metallic surface area (1.22 m^2^ g_cat_^–1^) and dispersion (18%), followed by Co–Ni/CeO_2_ (0.87 m^2^ g_cat_^–1^, 13%). Interestingly, simultaneous codeposition in NiCo/CeO_2_ resulted in lower values (0.74 m^2^ g_cat_^–1^, 11%) than sequential deposition, indicating that the synthesis strategy plays a decisive role in determining metal accessibility.

After observing the physical features of the catalysts, their reduction features were investigated by temperature-programmed reduction (Figure 2A). For the Ni/CeO_2_ catalyst, a shoulder at 185 °C and a main peak at 240 °C were observed, which can be attributed to the reduction of adsorbed oxygen and Ni species weakly interacting with the support and to the reduction of small NiO crystallites or Ni–O–Ce, respectively.^23^ In the Co/CeO_2_ catalyst, the peak at 305 °C corresponds to the reduction of Co^3+^ to Co^2+^, while the one observed at 430 °C corresponds to the reduction of surface oxygen in CeO_2_.^24^ The shoulder at 250 °C is probably related to the initial reduction of the Co with different oxidation states and dispersion. The bimetallic catalysts, however, displayed a comparable reduction profile characterized by a maximum at an intermediate temperature (285–290 °C), which can be related to Co reduction or Ni and Co coreduction after their interaction. In Co–Ni/CeO_2_, the peak at 430 °C is the reduction of surface oxygen in CeO_2_, suggesting that this catalyst presented a lower interaction with the support, as the Co/CeO_2_ catalyst.

**Figure 2:**
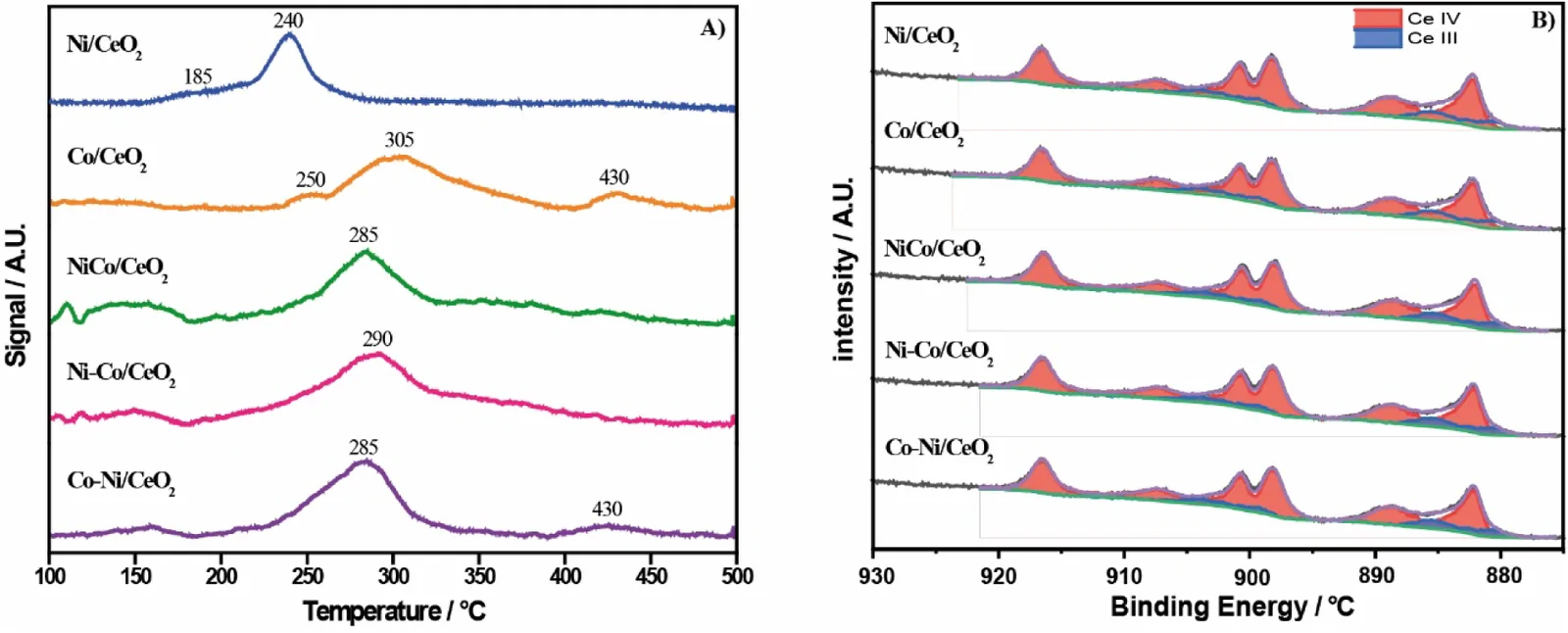
A) Temperature-programmed reduction profiles of the mono- and bimetallic catalysts. B) High-resolution XPS spectra for the prepared catalysts.

Finally, by using XPS (Figure 2B), further insights into the surface composition and chemical states of the catalysts were obtained. Although the metals’ signals were too weak or noisy for reliable quantitative analysis in the supported catalyst, the Ce 3d spectra for all materials showed the typical multiplet structure of Ce^4+^, accompanied by Ce^3+^ contributions.^21^ Fitting of the Ce 3d spectra was performed by the synthetic peak model approach, proposed by Morgan.^20^ All the samples presented approximately the same Ce^3+^ concentrations of 15–16%.

### 3.2 ESR Performance

The catalytic performances of the prepared materials were evaluated by ESR, and the results are presented in Figure 3. All catalysts showed high ethanol conversion at the beginning of the reaction but underwent different deactivation degrees in the following hours. Therefore, to compare them, their deactivation rate constant (kd) was calculated considering 6 h of reaction and first-order deactivation kinetics.

**Figure 3:**
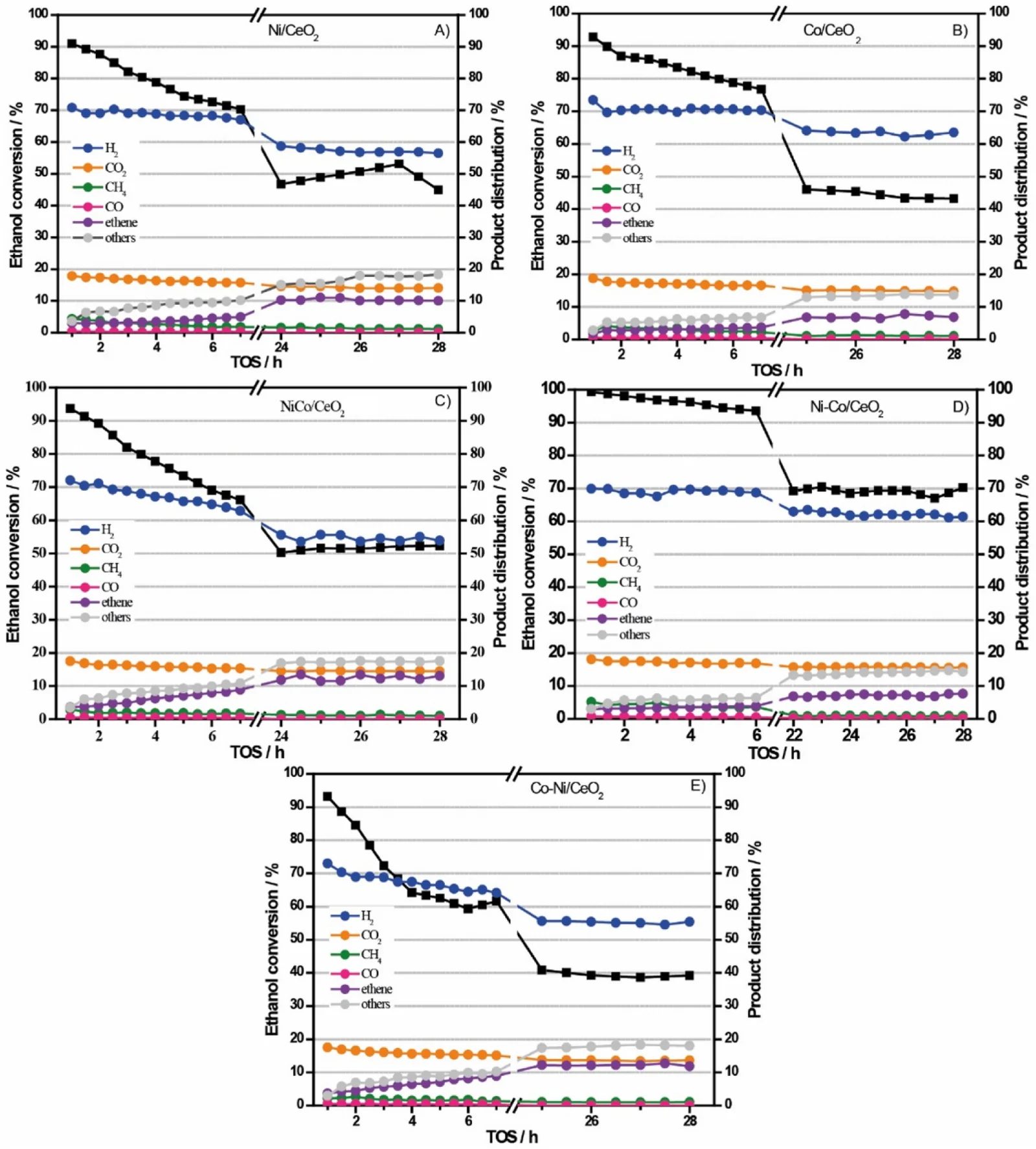
ESR performance of A) Ni/CeO_2_, B) Co/CeO_2_, C) NiCo/CeO_2_, D) Ni–Co/CeO_2_, and E) Co–Ni/CeO_2_ catalysts. The plot shows ethanol conversion (black squares, left axis) and product distribution (right axis) as a function of time on stream (TOS).

Both monometallic catalysts exhibited significant deactivation within the first 7 h of reaction, with ethanol conversion decreasing by 20% on Ni/CeO_2_ (kd = 3.77 × 10^–2^ h^–1^) and 10% on Co/CeO_2_ (kd = 2.72 × 10^–2^ h^–1^). The loss of activity was accompanied by a decline in H_2_ of 12% for the Ni/CeO_2_ and of 8% for the Co/CeO_2_ counterpart. Thereafter, the activity of both catalysts stabilized at ∼45% until the end of the 28 h test. The bimetallic catalysts showed distinct behaviors depending on the deposition order. Ni–Co/CeO_2_ (deposition of Co first) exhibited the highest activity and resistance to deactivation among the catalysts, presenting the lowest value for the deactivation rate constant (kd = 9.93 × 10^–3^ h^–1^). In addition, it maintained a high conversion (∼70%) until the end of the 28 h reaction. On the other hand, the NiCo/CeO_2_ (codeposition) and Co–Ni/CeO_2_ (Ni deposition first) catalysts showed the highest loss of activity with deactivation rate constants of 5.08 × 10^–2^ h^–1^ and 7.54 × 10^–2^ h^–1^, respectively. Furthermore, there was a decrease in reforming capacity by these catalysts, evidenced by a reduction in H_2_ selectivity and an increase in undesirable products.

In this sense, the sequential deposition of Co followed by Ni (Ni–Co/CeO_2_) resulted in the most balanced catalytic response, maintaining H_2_ selectivity near 63% with improved stability and lower levels of byproducts compared to the other bimetallic configurations. This behavior can be attributed to a Ni overlayer that effectively suppresses Co-driven decomposition reactions, thereby enhancing the reforming selectivity. Conversely, when the deposition order was reversed (Co–Ni/CeO_2_), the catalyst displayed reduced stability and lower hydrogen yields. Thus, the comparison demonstrates that while Ni is intrinsically more active and selective than Co, the bimetallic system with Ni deposited over Co provides the optimal compromise between hydrogen production and minimization of side products, underscoring the critical role of metal dispersion and the deposition sequence in determining ESR performance. Although the catalyst exhibited a lower degree of deactivation and the reduction in H_2_ selectivity was less pronounced compared to the other samples, and the explanations for this behavior are consistent, a deeper understanding of the underlying processes is still required to assess its overall performance reliably. Thus, characterization of the spent catalysts is mandatory.

### 3.3 Characterization of the Spent Catalysts

The carbon EDS mapping of the spent catalysts (Figure S3) provides complementary qualitative information about the spatial distribution of carbonaceous deposits over the catalyst surface. These maps are not intended to quantify the amount or determine the structural nature of the deposited carbon, which is more directly assessed by TGA-DTG and Raman spectroscopy. For the Ni/CeO_2_ sample (Figure S3A,B), the carbon signal is relatively distributed over the CeO_2_ surface, whereas Co/CeO_2_ (Figure S3C,D) exhibits more localized carbon-rich regions. Among the bimetallic catalysts, differences in the spatial distribution of the carbon signals are also apparent. NiCo/CeO_2_ (Figure S3E,F) shows substantial carbon coverage, while Ni–Co/CeO_2_ (Figure S3G,H), in which Ni was deposited after Co, exhibits a comparatively weaker and less extensive carbon signal. Co–Ni/CeO_2_ (Figure S3I,J), in which Ni was deposited before Co, displays the most pronounced and widespread carbon signal.

To complement these qualitative observations, thermogravimetric (TGA) and derivative thermogravimetric (DTG) analyses were carried out on the spent samples (Figure 4). The TGA curves (Figure 4A) reveal the weight losses associated with carbon combustion, which allow a comparative evaluation of coke content among the catalysts. The percentage of coke formation and coke production rate per gram of catalyst per hour in each material are available in Table 2. The results establish the following order of total coke deposition: Ni–Co/CeO_2_ < Ni/CeO_2_ ≈ Co/CeO_2_ < NiCo/CeO_2_ < Co–Ni/CeO_2_, in excellent agreement with the EDS mapping trends. Notably, the Co–Ni/CeO_2_ catalyst presents the largest mass loss, reflecting its high coke accumulation, while Ni–Co/CeO_2_ shows the lowest, being the most active catalyst. The DTG profiles (Figure 4B) further distinguish the nature of carbon species deposited. All catalysts display a main combustion event between ∼290 and 350 °C, typically assigned to nonstructured coke species. This amorphous coke covers the metal particles, preventing the adsorption of ethanol and water, which leads to the loss of catalyst activity. No peaks above 450 °C related to fibrillar coke oxidation were observed.

**Figure 4:**
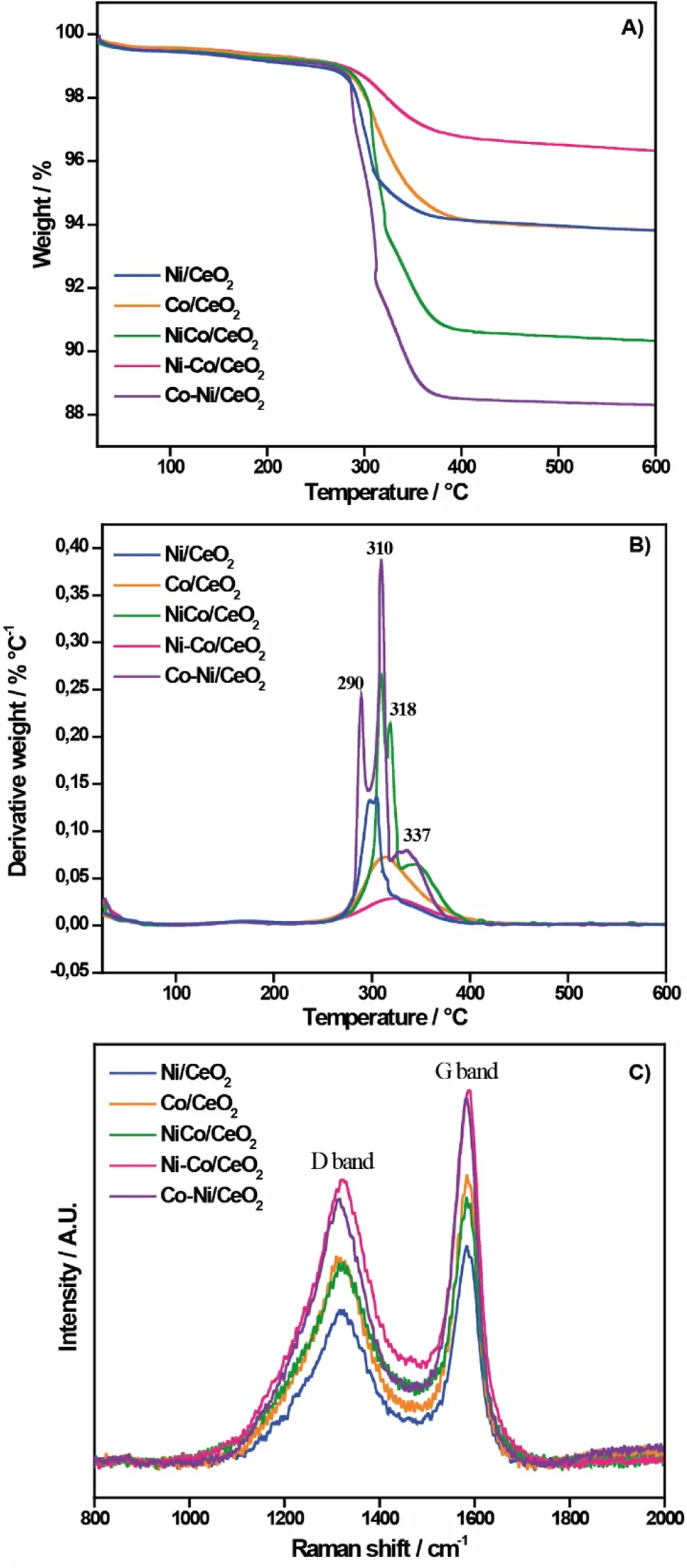
A) TGA, B) DTG profiles, and C) Raman spectra of the spent catalysts after the reaction.

**Table 2:** Characterization of the Spent Catalysts

	Raman spectroscopy	TPO analyses	
Material	I_D_/I_G_	Coke content (wt %)	mg_coke_ g_cat_^–1^ h^–1^
Ni/CeO_2_	0.66	6.4	2.3
Co/CeO_2_	1.24	6.5	2.3
NiCo/CeO_2_	0.69	10.0	3.6
Ni–Co/CeO_2_	0.74	4.0	1.4
Co–Ni/CeO_2_	0.70	11.9	4.3

Raman spectroscopy provides additional insights into the structural ordering of the deposited carbon (Table 2). In the Raman spectra of the spent catalysts (Figure 4C), the G band at 1580 cm^–1^ corresponds to the E_2_g vibrational mode of sp^2^ carbon, related to the vibration of C=C bonds in graphitic planes, and is associated with well-organized, more graphitic carbon structures with greater order. On the other hand, the D band at 1350 cm^–1^ indicates the presence of amorphous, nanocrystalline carbon, or carbon with many structural defects. For this reason, the ratio between the intensities of the D and G bands (I_D_/I_G_) is widely used as an indicator of disorder. High I_D_/I_G_ values indicate the predominance of disordered/amorphous carbon, while lower I_D_/I_G_ values suggest the formation of more graphitic and ordered carbon. Except for the Co/CeO_2_ catalyst, all materials presented very similar I_D_/I_G_ ratios; thus, considering such values and the DTG data, one can see that the nature of coke formation is similar among the samples; however, the synthetic protocol affected the quantity of their formation, affecting the performance and stability.

On the basis of the catalytic and postreaction characterization results, Ni–Co/CeO_2_ emerged as the most effective catalyst for ESR. This material exhibited the lowest deactivation rate constant, the highest stability, and the smallest decrease in H_2_ selectivity over 28 h of reaction. EDS mapping, TGA-DTG, and Raman analyses confirmed minimal coke deposition consistent with its sustained performance. These findings highlight the critical influence of the metal deposition sequence, as the configuration with Ni deposited over Co optimizes surface interactions, limits carbon accumulation, and enhances the reforming efficiency.

## 4 Conclusions

The present study demonstrated that low-load Ni- and Co-based catalysts (0.2 wt % total metal) prepared by magnetron sputtering onto nanocrystalline CeO_2_ exhibit distinct physicochemical and catalytic behaviors in ESR, with the observed differences depending on the metal deposition sequence. Structural and textural characterizations confirmed that the sputtering process afforded highly dispersed metallic species without the formation of large crystallites, while maintaining the mesoporous structure of the CeO_2_ support. Among the catalysts, Ni–Co/CeO_2_ (Co deposited first, followed by Ni) displayed the best catalytic performance, characterized by the lowest deactivation rate constant (kd = 9.93 × 10^–3^ h^–1^), high H_2_ selectivity (∼63%), and stable performance over 28 h of reaction. Postreaction analyses by EDS mapping, TGA-DTG, and Raman spectroscopy consistently revealed minimal coke formation on this catalyst, in contrast with the Co–Ni/CeO_2_ and NiCo/CeO_2_ counterparts, which suffered extensive carbon accumulation and faster deactivation. The higher metallic surface area and dispersion measured by H_2_ chemisorption for Ni–Co/CeO_2_ (1.22 m^2^ g_cat_^–1^ and 18%, respectively) likely contribute to its favorable catalytic behavior by increasing the accessibility of metallic sites. However, the differences between the sequentially deposited bimetallic catalysts indicate that metal accessibility alone may not fully account for the observed catalytic behavior, and the deposition sequence may also influence the local catalytic environment. Since the present STEM-EDS and XPS results do not directly establish the detailed nanoscale arrangement of Ni and Co, the proposed Ni-rich surface environment associated with the Co-followed-by-Ni deposition sequence should be regarded as a plausible interpretation of the catalytic and coke-formation results rather than as a demonstrated structural feature.

The findings also underscore the promising potential of magnetron sputtering as a synthesis approach for fine-tuning the surface composition and metal dispersion at very low loading levels. Even with only 0.2 wt % total metal, the catalysts exhibited distinct activity, selectivity, and stability, with Ni–Co/CeO_2_ showing the most favorable combination of these properties under the conditions investigated. Such results open possibilities for further investigations into the electronic structure and interfacial effects at higher metal loadings, aiming to deepen the understanding of the active site nature and optimize catalyst design for efficient hydrogen production.

## Supplementary Material



## Notes

Additional XRD, N_2_ adsorption–desorption, and SEM-EDS characterization data (PDF)

## ○

Divisão de Catálise, Biocatálise e Processos Químicos, Instituto Nacional de Tecnologia, Rio de Janeiro, RJ, 20081-312, Brazil
