# Supplementary material for: Toward More Cost-Effective Hydrogen Production: Magnetron-Sputtered Ni- and Co-Based CeO2 Catalysts at Ultralow Metal Loading
Source: ACS Omega. 2026 Oct 14;11(42):64956–64. doi: 10.1021/acsomega.6c06081 (PMC13717796; doi:10.1021/acsomega.6c06081)
Supplement: Supplementary file 1 [file ao6c06081_si_001.pdf]

Supporting information

Toward More Cost-Effective Hydrogen Production: Magnetron-Sputtered Ni- and Co-based CeO_2 Catalysts at Ultralow Metal Loading

Leticia Forrer Sosa[a,b], Marco Aurélio Suller Garcia[b,c], Adriano Friedrich Feil[d], Dario Eberhardt,[d] Santiago José Alejandro Figueroa,[e,f] Carlos Alberto Ospina Ramirez[g], Angela Albuquerque,[g] João Monnerat Araújo Ribeiro de Almeida,[a,b] Pedro Nothaft Romano[a,b,c,*]

[a]Instituto de Química, Universidade Federal do Rio de Janeiro (UFRJ), Rio de Janeiro 21941-909, RJ, Brazil.

[b]LIPCAT (Laboratório de Intensificação de Processos e Catálise), UFRJ, Rio de Janeiro 21941-594, RJ, Brazil.

[c]Nanotechnology Engineering Program, Alberto Luiz Coimbra Institute for Graduate Studies and Research in Engineering, COPPE, UFRJ, Rio de Janeiro 21941-972, RJ, Brazil.

[d]BlueNano, TECNOPUC, Porto Alegre 90610-001, RS, Brazil.

[e]Brazilian Synchrotron Light Laboratory (LNLS), Centro Nacional de Pesquisa em Energia e Materiais (CNPEM), 13083-100, Campinas, SP, Brazil.

[f]Institute of Chemistry, University of Campinas, Campinas, SP, 13083-862, Brazil.

[g]Brazilian Nanotechnology National Laboratory, Brazilian Center for Research in Energy and Materials, Campinas, SP, 13083-100, Brazil.

* pedro.romano@caxias.ufrj.br

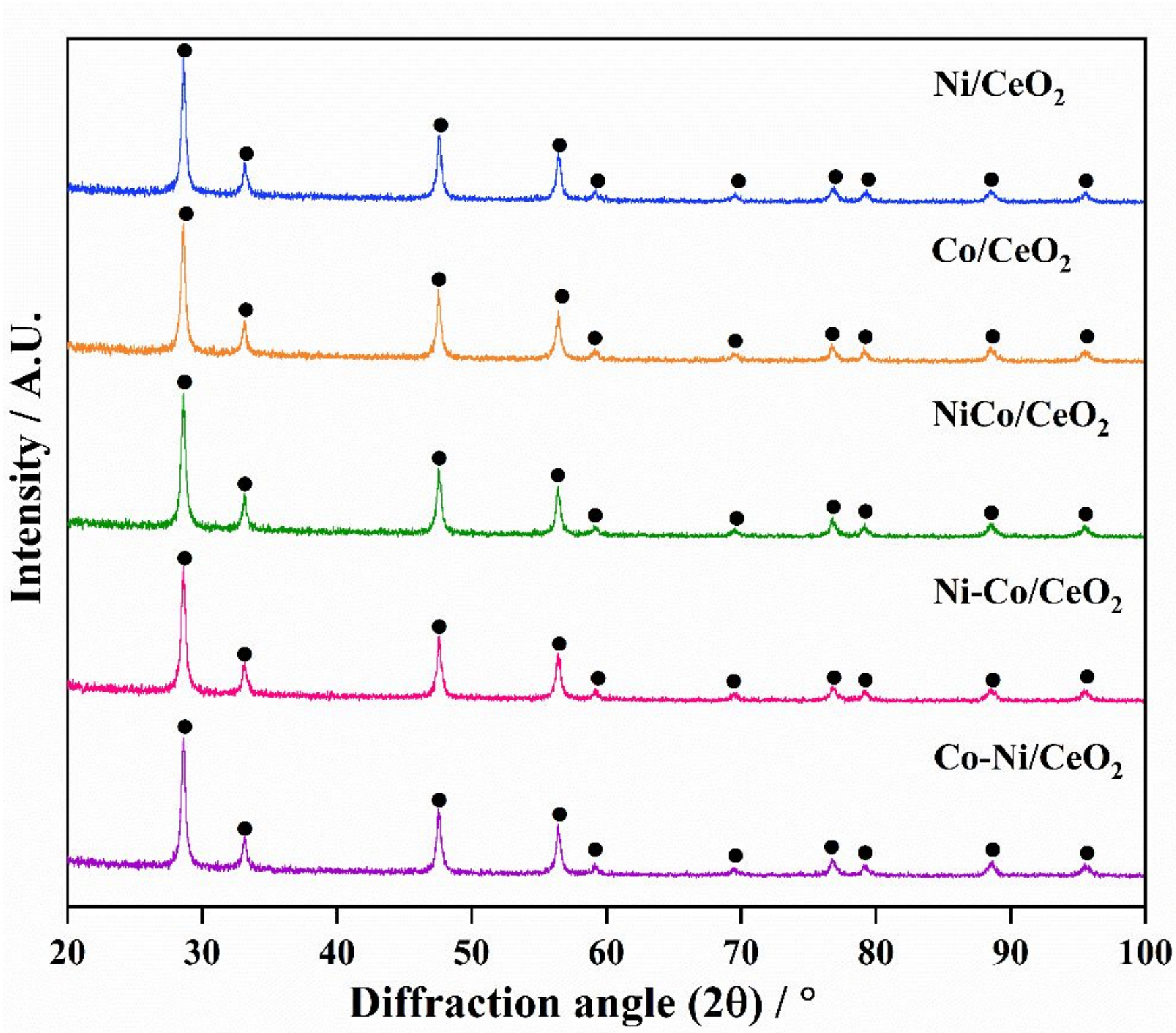

Figure S1. Diffractograms of mono- and bimetallic Ni/Co catalysts. ● CeO_2.

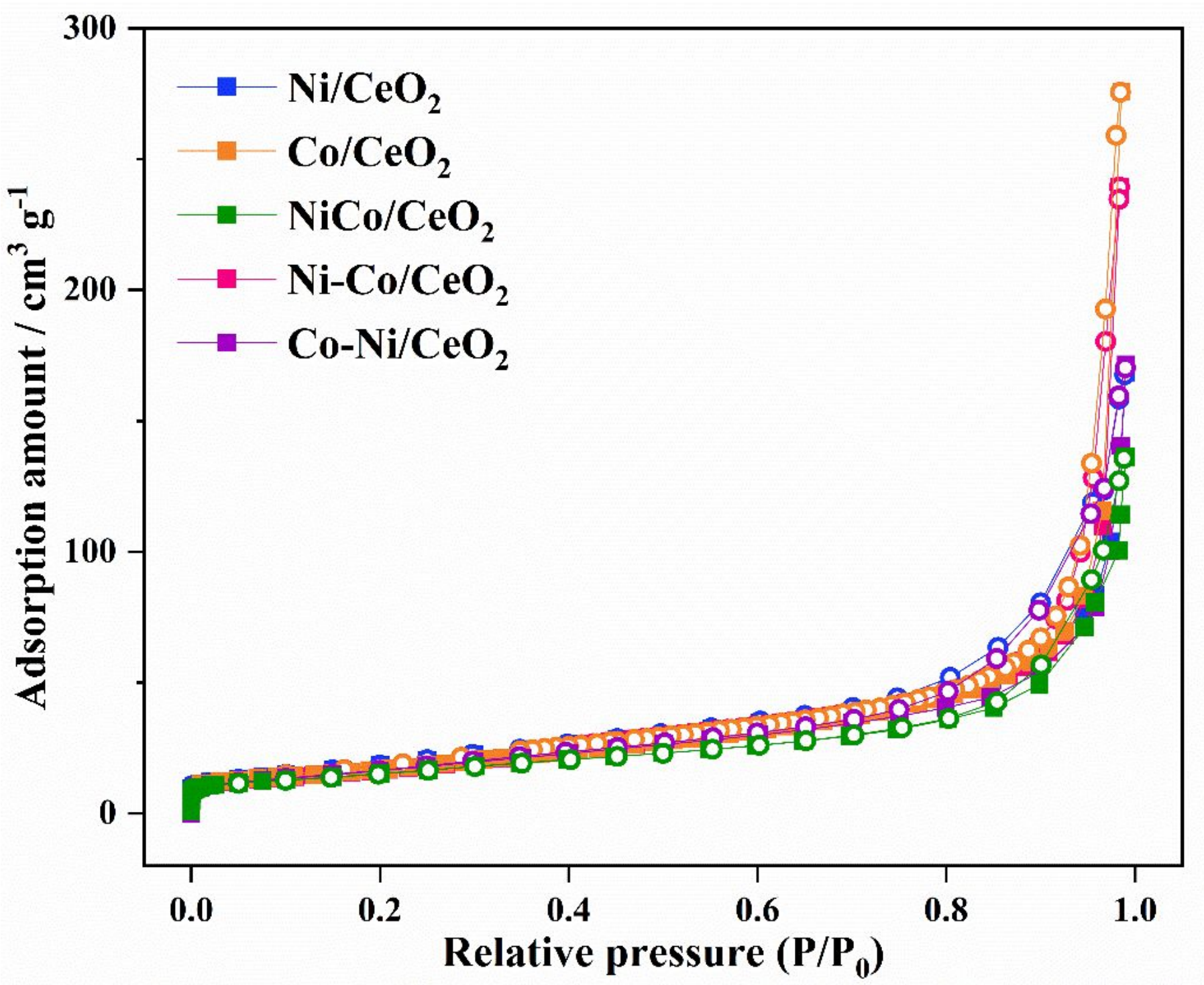

Figure S2. N_2 adsorption-desorption isotherms of mono- and bimetallic catalysts.

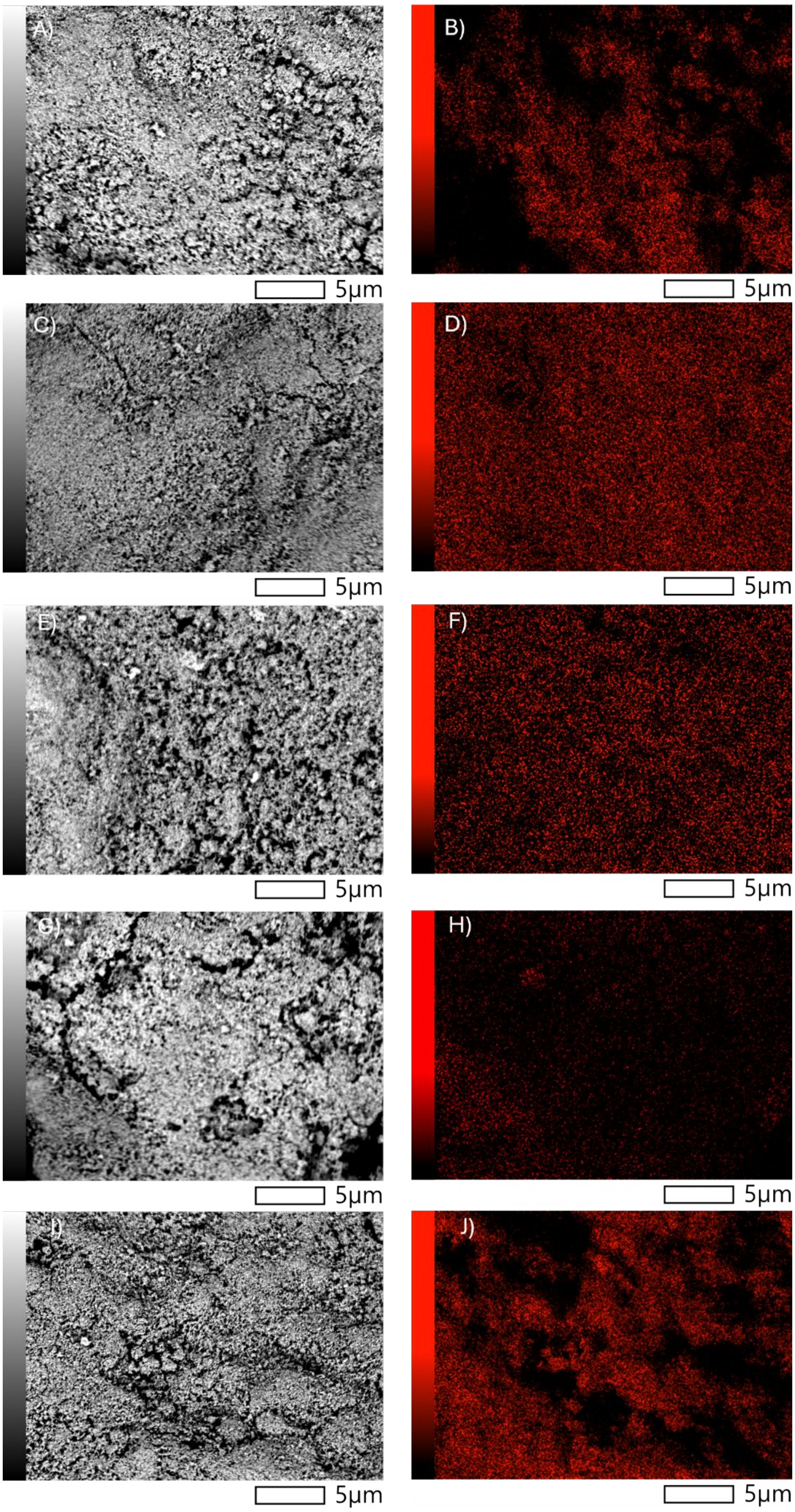

Figure S3. SEM micrographs (left) and corresponding carbon EDS mapping (right) of spent catalysts after ESR: A-B) Ni/CeO_2, C-D) Co/CeO_2, E-F) NiCo/CeO_2, G-H) Ni-Co/CeO_2, and I-J) Co-Ni/CeO_2.
